# Bridging the Translational Divide in Pain Research: Biological, Psychological and Social Considerations

**DOI:** 10.3389/fphar.2021.603186

**Published:** 2021-04-15

**Authors:** Chulmin Cho, Harashdeep K. Deol, Loren J. Martin

**Affiliations:** Department of Psychology, University of Toronto Mississauga, Mississauga, ON, Canada

**Keywords:** pain, translation, memory, mouse, social, biopsychosocial

## Abstract

A gap exists between translating basic science research into effective pain therapies in humans. While preclinical pain research has primarily used animal models to understand biological processes, a lesser focus has been toward using animal models to fully consider other components of the pain experience, such as psychological and social influences. Herein, we provide an overview of translational studies within pain research by breaking them down into purely biological, psychological and social influences using a framework derived from the biopsychosocial model. We draw from a wide landscape of studies to illustrate that the pain experience is highly intricate, and every attempt must be made to address its multiple components and interactors to aid in fully understanding its complexity. We highlight our work where we have developed animal models to assess the cognitive and social effects on pain modulation while conducting parallel experiments in people that provide proof-of-importance for human pain modulation. In some instances, human pain research has sparked the development of novel animal models, with these animal models used to better understand the complexity of phenomena considered to be uniquely human such as placebo responses and empathy.

## Introduction

In order to completely understand complex conditions such as chronic pain, multiple factors that contribute to the personalized experience of pain must be considered. The biopsychosocial model, introduced by (Engel 1977), emphasizes the importance of considering biomedical evidence along with a patient’s subjective psychological wellbeing and social environment in the manifestation and progression of an illness. Though biological components such as genetics may predispose individuals to certain chronic pain conditions, psychological and social variability often impact the time of onset, severity and the course of pain associated with multiple conditions (Engel 1981). Moreover, psychological and social disturbances can often influence overall health and exacerbate existing illness-related symptoms (Engel 1981). For example, we naturally assume that people with high levels of pain and disability always must have had more severe injuries than those who have less pain and disability. Of course, sometimes this is true, but in the majority of cases, psychological influences such as anxiety, fear and even social relationships can imbue either a positive or negative effect upon one’s pain perception and experience (Salovey et al., 2000; Pressman and Cohen 2005; Pulvers and Hood 2013).

The biopsychosocial model encompasses biological, psychological, social and all other associated processes that include cognitive, affective and behavioral constructs (Meints and Edwards 2018). It is precisely these biopsychosocial influences that are rarely considered when novel drugs are screened using preclinical models and eventually assessed in clinical trials. By only evaluating biological contributors, we are missing a fundamental aspect of the pain experience and the lack of psychological and social considerations may hinder understanding chronic pain conditions in their entirety. Herein, we parse out the biopsychosocial framework into distinct lines of pain research that consider biological, cognitive, psychological and social influences. We begin by discussing common biological approaches and our own research that identified genes associated with the development of temporomandibular joint disorder pain in people and then used animal models to understand their involvement in pain processing and identify a biological mechanism (Martin et al., 2017). In separate lines of research, we have used unconventional approaches and formed novel collaborations with psychologists and social scientists to perform contemporaneous studies where virtually the same experiment is conducted in mouse and human subjects. To this end, we have investigated acute pain modulation as an aberrant memory and have developed novel models to examine the memory of pain in both mice and people (Martin et al., 2019). Our work has also highlighted the importance of the social context by studying empathy by using mouse and human subjects (Martin et al., 2015). The “pain memory” and “empathy” themes assume that a significant contributor to pain modulation and chronification is the context through which we experience pain.

## Biological Considerations

Animal models–comprising the bulk of preclinical pain research–have received a lot of criticism for their lack of clinical validity. Yet, these same animal models provide the blueprints for drug development and ultimately failed clinical trials (Bennett and Xie 1988; Wallace et al., 2002; Mogil et al., 2010). Over the last few decades, only a handful of new and highly efficacious analgesics have been developed by the pharmaceutical industry despite identifying hundreds of novel molecular targets and investment of billions of dollars (Kissin 2010; Woolf 2010). However, the majority of analgesics developed within the past five years are mostly conjugates of existing drugs, reformulations or chance observations that have led to the repurposing of drugs designed to treat another disease (Smith et al., 2013; Moore et al., 2015; Sisignano et al., 2016). The failure of animal models has led many in the pain field to re-evaluate preclinical animal models and ask how the preclinical research process may be improved. Several labs now incorporate tissue samples from animal and human donors to understand where similarities and differences may exist (Ray et al., 2018; Sheahan et al., 2018; Dedek et al., 2019). The validation of new molecules by using human tissue is advantageous because confidence in targets increases before progressing toward much more time-intensive and costly clinical trials. However, this approach still does not adequately address what biological substrate–molecules, cells, circuits or systems–should be prioritized to produce the most effective pain therapies.

The development of anti-CGRP (calcitonin gene-related peptide) antibodies for the treatment of migraines is a recent example of translational success. The successful development of these drugs was based on a detailed understanding of CGRP signaling, key clinical evidence for the role of CGRP in migraine headaches and a precise role for CGRP in the trigeminovascular system in the pathophysiology of migraine (Edvinsson et al., 2018). The evidence was too overwhelming for anti-CGRP-based pharmaceuticals not to work as migraine therapeutics; however, this was not an overnight success as CGRP was discovered in 1982 and has been extensively studied ever since. In stark contrast, one of the most devastating failures of pharmaceutical translation was the neurokinin 1 (NK1) receptor antagonists from rodents to humans. Despite an overabundance of research focused on the role of substance P in pain transmission, the development of high affinity and selective NK1 receptor antagonists and the plethora of animal data supporting their utility as analgesics, the clinical trial data did not support the profile of these drugs as effective analgesics in a variety of pain states (Boyce and Hill 2004). In clinical trials, NK1 receptor antagonists failed to show any analgesic efficacy in patients suffering from migraines, visceral pain, osteoarthritis and fibromyalgia (Borsook et al., 2012). The differences observed between animals and humans may not only be explained by species differences in substance P/NK1 receptor but also by the lack of understanding of how NK1 receptors function and vary across species (Hill, 2000; Khan, 2015; Navratilova and Porreca, 2019). In the case of NK1 receptors, their activation induces a redistribution to endosomes that causes sustained excitation of spinal neurons and pain transmission, which is alleviated by targeting endosomal NK1 receptors. Thus, conventional NK1 receptor antagonists as chronic pain therapies may have failed due to their inability to inhibit internalized NK1 receptors (Jensen et al., 2017). The presumption that animal models recapitulate the disease being studied or the hypothesis being pursued can often constrain research. Such assumptions may cloud the interpretation of scientific findings which can often result in the failure of possible drug treatments and therapies in clinical trials.

To improve translation between humans and animal models, we and others have reversed this process. Specifically, we used human genetics to identify unique single nucleotide polymorphisms in genes associated with clinical pain and then used animal models to understand how they regulate pain and their involvement in chronic pain (Martin et al., 2017). Our human genetics studies identified polymorphisms in the genes encoding for epiregulin (EREG) and the epidermal growth factor receptor (EGFR) as among the top three “hits” associated with the development of clinical pain. EREG and EGFR form a ligand and receptor, respectively, that are well-studied in cancer research, yet their prominent role in pain processing remained unclear. Albeit, a few case reports had shown that EGFR inhibitors may provide pain relief in cancer patients (Moryl et al., 2006; Kersten and Cameron 2012) and neuropathic pain patients (Kersten et al., 2015). There was also evidence that serum concentrations of epiregulin and other EGFR ligands are upregulated in rheumatoid arthritis patients. Local blockade of these growth factors suppressed the development of cytokine-induced arthritis in mice by inhibiting chemokines and interleukin-6 expression (Harada et al., 2015). These studies, combined with our genetic association results, gave us confidence in fully exploring the contribution of EGFR and EREG to nociception using animal models. We found that inhibiting EGFR at the tyrosine kinase site reduced nociceptive sensitivity in mouse models of inflammatory and neuropathic hypersensitivity, including spontaneous facial expressions (Martin et al., 2017). Further, epiregulin administration increased inflammatory nocifensive behavior through a mechanism involving the PI3K/AKT/mTOR pathway and matrix metalloproteinase 9 in sensory neurons (Martin et al., 2017).

Currently, available EGFR inhibitors are associated with unwanted side effects, including pruritus (i.e., itchy skin rash) and severe diarrhea^7^. These side effects would limit their utility as analgesics. This is where further work using animal models would be necessary and may help develop and refine EGFR-based therapies devoid of side effects that would preclude their use by pain patients. We recently showed that a monoclonal antibody targeting epiregulin reversed inflammatory nociception. Still, the timing of antibody administration was critically important in determining whether nociceptive reflexes were augmented or attenuated in mice (Verma et al., 2020). Together, our mouse studies combined with clinical observations and small-scale clinical trials showing the efficacy of EGFR inhibitors for the treatment of refractory pain conditions provide compelling evidence for their utility as a novel analgesic strategy. The aforementioned epiregulin antibody experiments used mice but were informed by clinical human genetic association data showing that patients with different polymorphisms of the EGFR recover differently. The next step would be to develop a humanized version of this antibody for small-scale testing in people. Although, the development of novel pain therapies such as EGFR inhibitors or antibodies should more comprehensively assess the pain experience through the incorporation of a wide spectrum of pain behavior including memory, conditioning, and affective consequences.

In our studies, EGFR polymorphisms were identified in the white blood cells of chronic pain patients. We used this finding as a proxy to investigate EGFR-related signaling in sensory neurons (i.e., dorsal root ganglion, DRG); however, this approach was not guaranteed to work, and EGFR-related pain modulation may have occurred within the skin or at some other site. These alternatives could have easily been investigated by directly comparing the same site of innervation (DRG, spinal cord, brain) using rodent models and human samples, typically taken from post-mortem donors (Ray et al., 2018; Sheahan et al., 2018; Dedek et al., 2019). While the upregulation of DRG-specific ion channels in mouse and rat models of neuropathic pain (LaCroix-Fralish et al., 2011; Zhang and Dougherty 2014) and human neuropathic pain (Li et al., 2018) has been observed, transcriptome analysis indicates the presence of stark sex differences for neuropathic pain in both species for the gene modules and signaling pathways associated with immune responses and neuronal plasticity (North et al., 2019; Stephens et al., 2019). Evidence toward sexually dimorphic mechanisms of ion channel regulation in pain has found higher expression of some ion channels including ANO8, GRIK5, GRIN1, HCN2, KCNAB2, KCNC1, KCNG1, KCNH2, KCNK3, and PANX2 in a male-pain cohort compared to a female-pain cohort. However, it is essential to recognize that baseline differences in gene expression between the sexes could account for a certain degree of differential expression (Stephens et al., 2019). Animal models are highly valuable in this respect because differential gene expression can be directly controlled through comparisons with naïve animals. Still, species considerations are much more difficult as the abundance of ion channels and receptors involved in pain processing may be vastly different between species (Shiers et al., 2020).

To completely understand the involvement of specific genes, follow-up studies using a candidate approach must be undertaken. A candidate approach could be in the form of a genetic knockout or knockdown study using animal models, potential transgenic “rescue” experiments and the incorporation of pharmacological tools to block proteins of interest. For instance, studies have confirmed sex differences in pain modulation, including the genetic variation of *OPRK1* (encoding κ-opioid receptor) in humans (Gear et al., 1996; Sato et al., 2013) and the efficacy of κ-opioids to induce analgesia in rats with female rats exhibiting quicker analgesic onset and magnitude (Bartok and Craft 1997). Strikingly, the sex-dependent response of females to *κ*-opioid agonists may not be directly related to OPRK1, but instead the melanocortin-1 receptor gene (*MC1R*). Mogil et al., 2003 used quantitative trait locus mapping, a candidate gene strategy and pharmacological tools to reveal that the *MC1R* mediates *κ*-opioid analgesia in female mice only. Their translational studies showed that women with variations of the *MC1R* allele, associated with red hair and fair skin, also display greater analgesia from the *κ*-opioid pentazocine. However, in humans, subjects’ genetic characteristics must be measured rather than assumed. For example, resistance to subcutaneous lidocaine was measured in women with red hair that were assumed to have the mutated *MCR1* gene even though genetic mutations in *MCR1* were not directly measured (Liem et al., 2005). The same group has also shown that genetic mutations in *MCR1* are associated with an increased requirement for general anesthesia in redheads (Liem et al., 2004).

Both clinicians and experimentalists report that sensitivity to pain, propensity to develop painful pathology and response to pain-inhibiting (i.e., analgesic) strategies all feature large individual differences (Elmer et al., 1998; Nielsen et al., 2008; Nielsen et al., 2009). The genetic portion of such variability can be studied using inbred mouse strains and analogous twin studies in humans (Mogil et al., 1999; Lariviere et al., 2002; Smith et al., 2004; Fillingim et al., 2008). However, these types of heritability studies have made it clear that most of the observed variance–even in the laboratory environment–is not explained by genetic factors, but environmental influences and their interaction with genes (Mogil et al., 2004). In mice, a within-cage “order-of-testing effect” in which the first mouse in a cage tested on the tail-withdrawal test displays higher withdrawal latencies (i.e., lower sensitivity) than subsequently tested mice from that cage (Chesler et al., 2002a; Chesler et al., 2002b). We have also shown that the gender of the experimenter impacts pain behavior in mice (Sorge et al., 2014), while others have observed a similar phenomenon for human pain testing (Wise et al., 2002; Gijsbers and Nicholson 2005). Several laboratory environmental factors are also known to influence baseline nociceptive responding in mice, including housing, diet, test conditions and experimental design (for a full review see Mogil 2017). Certain aspects of these phenomena may be driven by epigenetic factors, which are known to cause changes in memory (Chwang et al., 2007), nociceptive sensitization (Chiechio et al., 2010) and behavioral responses to opioid-based drugs (Liang, Li et al., 2013). Research in epigenetics of pain has revealed that changes within an individual’s environment can lead to heritable changes in gene function through processes like histone modification, DNA methylation and chromatin remodeling (Denk and McMahon 2012; Crow et al., 2013), all of which may influence replication between laboratories and translation.

## Cognitive and Psychological Considerations

The perception of pain, whether acute or chronic is a subjective experience modulated by our history and expectations (Flor 2002). To complicate matters further, individual differences in the perception of the environment despite the same physical stimuli led to vastly different pain experiences (Tabor et al., 2013; Harvie et al., 2016). In a remarkable study, Moseley and Arntz (2007) placed a cold piece of metal on the hand of subjects for 500 ms and asked subjects to rate their pain when shown either a red or blue visual cue. These were healthy participants who were not told why they were being shown the light, it was just part of the context and coincided with the application of the cold stimulus. Amazingly, for some people, pain was rated as more intense when a red light was shown as opposed to a blue light even though the nociceptive stimulus in both conditions was identical—the reason being that the evaluative context was critical for modulating the pain experience. Throughout our lives, we have learned to associate the color red with hot and potentially dangerous situations, while blue is typically associated with cool, calm and less damaging stimuli. Observations such as these have led others to investigate the individualized expression of pain, which is, in part, influenced by the emotional context such as motivation, arousal, mood, and learning (Miron-Shatz et al., 2009; Murty et al., 2010; Mirandola and Toffalini 2016).

The seminal work by Fordyce (Fordyce et al., 1973) brought into light the significance of learning in chronification of pain and its treatment. Fordyce postulated that the pain response in chronic pain is a learned behavior that can be altered using learning mechanisms such as operant conditioning. In operant conditioning, an association is formed between a (pain) behavior (e.g., verbalizations, actions and facial expressions) and the consequence of that behavior in the form of positive or negative reinforcement. In line with this, patients with chronic musculoskeletal pain increased their pain behavior when it was reinforced with positive reinforcements (i.e., attention and empathy) by the caregivers (Romano et al., 2000). Conversely, operant conditioning was used to mitigate pain behavior of chronic pain patients with musculoskeletal pain and fibromyalgia by reinforcing positive behaviors and extinguishing negative behaviors (Fordyce et al., 1973; Thieme et al., 2003; Thieme et al., 2006). Operant conditioning was also effective in healthy individuals using pressure (blood-pressure cuff) (Jolliffe and Nicholas 2004), heat (Hölzl et al., 2005; Kunz et al., 2011) and electric stimuli (Lousberg et al., 2005). This change in behavior brought on by operant conditioning elicited changes in pain-related somatosensory evoked brain potentials and are thought to involve the anterior cingulate cortex and the primary and secondary somatosensory cortices (Flor et al., 2002), but the underlying mechanisms remain largely elusive.

In laboratory animals, it is difficult to isolate and recapitulate operant conditioning as behavioral assays often involve both operant and classical (Pavlovian) conditioning (Li, 2013). However, efforts have been made to develop operant measures to analyze the affective component of pain in animals (for review see Navratilova and Porreca, 2014). One such measure is conditioned place preference (CPP), which has traditionally been considered to be dependent on a classical conditioning mechanism, but is in part, an operant measure of the affective component of pain (Huston et al., 2013). CPP was used to demonstrate in male rats with an incisional paw injury that local analgesia increases a rat’s preference toward a chamber associated with pain relief (Navratilova et al., 2012). Physiologically, this motivational behavior was driven by activation of ventral tegmental dopaminergic cells and increased release of dopamine in the nucleus accumbens, which supports the hypothesis that analgesia induces negative reinforcement—elicited by relief of an aversive state—via the mesolimbic reward pathway (Navratilova et al., 2012).

Accumulating evidence suggests that pain perception is shaped by our prior experience with pain and its relief, occurring through the creation or erasure of memory traces in peripheral neurons, the spinal cord and the brain (Ji et al., 2003; Sandkuhler and Lee 2013; Price and Inyang 2015). The term “pain memory” was coined by Dennis and Melzack (Dennis and Melzack 1979) following the observation that exposure to painful irritation of the forepaw in male rats before denervation of dorsal roots in the spinal cord (rhizotomy) led to acceleration and exacerbation of the neuropathic pain. They postulated that the pre-injury irritation created a pain memory that became disinhibited once the descending inhibitory control system was disconnected due to rhizotomy. The findings here can be translated to patients suffering from phantom limb pain who retain somatosensory memories about their pain before the amputation and still experienced the pain sensation following the amputation (denervation) (Katz and Melzack 1990; Flor et al., 2006). With somatosensory memory, many amputees retain a phantom limb's sensation being immobile and in the same position as before its amputation (Katz and Melzack 1990). Therefore, in part, phantom limb pain may derive from a proprioceptive memory mechanism where patients retain memory engrams of the limb before amputation (Gentili et al., 2002).

Physiologically, these long-lasting pain memories manifest as alterations of both the peripheral and central nervous systems (Flor et al., 2006). Peripherally, increased C-fiber afferent activity has been linked with pain sensation in amputees (Nyström and Hagbarth 1981). This finding was complemented in animals, where blocking C-fiber afferents in male rats with nerve injury attenuated hyperalgesia (Coderre and Melzack 1987). Local nerve block had a similar effect in patients with phantom limb pain where blocking the affected dorsal root ganglion led to immediate pain relief (Vaso et al., 2014). Centrally, among other changes, somatosensory pain memory elicits topographically reorganization of the primary somatosensory cortex (SI) (Ramachandran et al., 1992; Elbert et al., 1994; Halligan et al., 1994; Flor et al., 1995; Doetsch 1998). In animals, the amputation of digits in monkeys led to a reorganization in SI, where the representation of deafferented fingers was taken over by adjacent areas (Merzenich et al., 1984). In dorsal rhizotomy, the extent of SI reorganization was found to be even greater than the amputation of digits (Pons et al., 1991). Stimulation of the SI cortex has been found to induce phantom limb pain, and removal of this region ameliorated phantom limb pain (Head and Holmes, 1911; Appenzeller and Bicknell 1969), demonstrating the importance of somatosensory pain memory in phantom limb pain. Further research is still needed regarding the molecular determinants of pain memory in the context of phantom limb pain and longitudinal studies examining the relationship between pain experience and phantom limb pain.

When it comes to pain memory, children are highly vulnerable to behavioral and perceptual alterations induced by pain experience. Given the malleability of pain memories, childhood presents an opportunistic window to target pain management interventions to mitigate long-term consequences (Noel et al., 2017). Several studies have found that children who remember the nociceptive stimulus as worse than their initial experience rated the pain as greater when re-exposed to the same nociceptive stimulus (Chen et al., 2000; Noel et al., 2012; Noel et al., 2017). Interestingly, in children, pain memory served as a strong predictor of subsequent pain ratings (Noel et al., 2012; Noel et al., 2017). These findings in children are, in part, complemented by animal studies where neonatal injuries were associated with changes in nociceptive sensitivity. In rat neonates, a skin-deep injury to the hind paw led to increased sensitivity to mechanical stimuli three weeks following injury, indicative of long-term hypersensitivity (Reynolds and Fitzgerald 1995). In another study, rat neonates administered an inflammatory stimulus (i.e., complete Freund’s adjuvant) showed long-term alterations in pain response during adulthood facilitated by increased spinal circuit input and sprouting nociceptive primary afferent axons (Ruda et al., 2000). Furthermore, rat neonates exposed to colorectal distension led to colon hypersensitivity in adulthood (Al-Chaer et al., 2000). Neonatal injuries in rats also led to hypoalgesia, or reduced nociceptive sensitivity, when exposed to repeated formalin injections and tested for thermal sensitivity in adulthood (Bhutta et al., 2001). Still, in its infancy, similar findings in children and neonates encourage expanding the study of pain memory during development into various species and verifying their validity for translational purposes.

**FIGURE 1:**
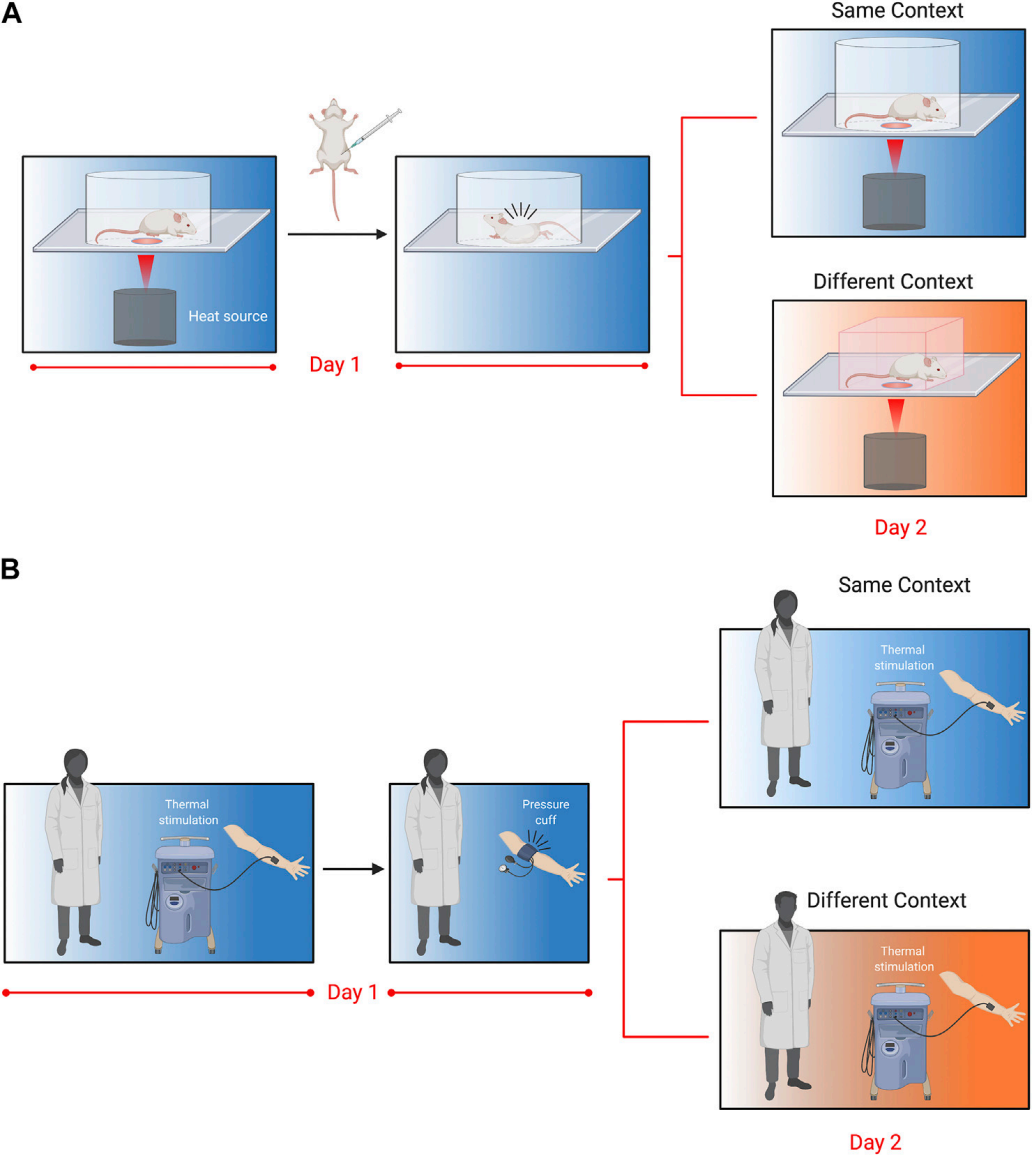
Translational behavioral models to test pain memory in mice and humans as originally reported in Martin et al., 2019. **(A)**. Mice are placed in Plexiglas cylinders and paw withdrawal thresholds evoked by thermal stimuli are measured every 5 min for 30 min. Following baseline measurements, mice are injected with acetic acid (i.p.,; 0.9%), which causes abdominal cramps and nociceptive writhing behavior that lasts for approximately 30 min. Twenty-four hours later, mice are placed either back in the same cylinder in the same room, or in a novel cubicle in a different room, and again tested for thermal withdrawal latencies. Nociceptive sensitivity was increased in mice returned to the same context 24 h following acetic acid injection, but this was only observed in male mice. Follow-up experiments revealed that enhanced nociceptive sensitivity in males was dependent on testosterone, the stress response and atypical PKCs. **(B)**. In the human model, participants were tested for their thermal sensitivity to a heat probe placed on the volar aspect of the forearm. Volunteers were then subjected to a submaximal effort ischemic tourniquet test for 20 min. Twenty-four hours later, participants were tested for thermal sensitivity in the same room by the same experimenter, or a different room (in a different building) and a different experimenter. Participants returned to the same room rated the thermal pain as higher, but this was only observed in men and associated with their pre-test stress response.

We have recently developed a novel assay to assess pain memory more directly in mice (Figure 1) (Martin et al., 2019). This behavioral model exploits prior pain experience to alter nociceptive sensitivity and examine the influence of environmental variables. In brief, we exposed mice to a specific context and administered an acute visceral nociceptive stimulus (i.e., acetic acid) that lasted for approximately 30 min (Figure 1A). When mice were re-exposed to the same context 24 h later, mice displayed hypersensitivity to a thermal stimulus. The working model is that exposure to nociceptive stimuli act as unconditioned stimuli leading mice to develop a negative association with their environment. In turn, this increased their nociceptive sensitivity (when returned to that environment), a phenomenon that we refer to as *conditioned pain hypersensitivity*. Interestingly, conditioned hypersensitivity was observed exclusively in male mice, and was context specific. This model does not seem to reflect human-like nocebo phenomena because proglumide (1 and 10 mg/kg), a cholecystokinin receptor antagonist that blocks nocebo responses in people failed to block conditioned pain hypersensitivity in mice (*unpublished observations*). It also does not represent general priming of the pain system because the context where the pain occurred is critical, indicating that memory of the environment is necessary for the observed hypersensitivity. Physiologically in males, testosterone was important as castration, and blocking testosterone abolished the conditioned pain hypersensitivity. Furthermore, atypical protein kinase C (aPKC), which includes the protein kinase Mζ isoform (PKMζ), was found to be critical at both spinal and brain level as inhibition prevented conditioned hypersensitivity. Given PKMζ’s postulated role in synaptic long-term potentiation and maintenance of long-term memory (Sacktor and Hell 2017), this finding adds to the accumulating evidence for the existence of pain memory and its effects on pain sensitivity.

The translational potential of our conditioned pain hypersensitivity model is exceptionally high because of contemporaneous experiments we conducted in humans that showed an excellent congruence with the mouse data (Figure 1B). Similar to the mouse experiments, healthy participants were exposed to the ischemic tourniquet procedure (i.e., unconditioned nociceptive stimulus) in a specified context. The next day participants returned for a second experimentation day. They were either tested by the same experimenter in the same room (i.e., same context) or a different experimenter in a different building (i.e., different context). Males tested in the same context on the second day reported increased ratings of pain intensity. This pain hypersensitivity was associated with increased stress ratings in males tested for pain sensitivity in the same context, and similar to mice was absent in female participants. These results showed a surprisingly direct translation of context-dependent conditioned pain hypersensitivity between the two species (Martin et al., 2019).

The studies outlined here highlight the widespread nature of pain memory and add a layer of complexity to studying pain modulation and mechanisms. On the other hand, this presents us with a unique challenge where novel strategies may be developed to improve pain outcomes by focusing on pain memory to yield positive symptom relief through learning. This is highlighted by placebo analgesia, a phenomenon where an inert treatment produces an improvement in symptoms (Levine et al., 1978). Cognitively speaking, two principal theories were developed as the basis for the activation of placebo analgesia: the expectation of pain relief and learning symptom relief by the repeated association between an active analgesic and therapeutic context (i.e., classical conditioning) (Amanzio and Benedetti 1999). One of the first studies to demonstrate placebo analgesia through conditioning was by Voudouris et al., 1989. In this study, participants were conditioned by repetitive pairings of a neutral non-anesthetic cream with electrical stimulation below their noxious threshold. Following conditioning, participants formed an associative memory between the placebo cream and the reduced electrical stimulation, which resulted in placebo analgesia to a higher intensity of electrical stimulation. Since then, numerous laboratories have successfully demonstrated placebo analgesia using classical conditioning (Amanzio and Benedetti, 1999; Price et al., 1999; Colloca and Benedetti, 2006; Klinger et al., 2007; Jensen et al., 2012). It should also be noted that conditioning produced a more substantial placebo effect than those induced with expectation (i.e., verbal cues and suggestion) (Colloca et al., 2008). Mechanistically, expectation-induced placebo analgesia is mediated mainly by the endogenous opioid system and is reversible by administering the opioid antagonist naloxone (Amanzio and Benedetti, 1999). However, conditioning-induced placebo analgesia can activate different subsystems depending on the unconditioned stimulus. For example, pharmacological conditioning using morphine engages the opioidergic system, while conditioning using ketorolac, a non-steroidal anti-inflammatory drug (NSAID), activates a non-opioid-based mechanism. While the former is blocked by naloxone, the latter is dependent on the endogenous cannabinoid system and blocked by rimonabant, a cannabinoid receptor type 1 blocker (Amanzio and Benedetti, 1999; Benedetti et al., 2011). These findings are complemented by brain imaging studies that have demonstrated that placebo analgesia is mediated by functional changes in cortical, subcortical and brainstem structures (Petrovic et al., 2002; Wager et al., 2004; Zubieta et al., 2005; Bingel et al., 2006; Eippert et al., 2009a). These include pain and affect processing regions such as the anterior cingulate (ACC), insula, thalamus, hypothalamus, periaqueductal gray (PAG) and rostral ventromedial medulla (RVM) (Wager et al., 2004; Eippert et al., 2009a; Atlas and Wager, 2014) and other higher-order regions that include dorsolateral prefrontal cortex (DLPFC) as well as the nucleus accumbens, which mediates reward behavior (Zubieta et al., 2005). Reduced activity in the spinal cord has also been observed (Eippert et al., 2009b). Furthermore, functional brain imaging studies have identified changes within brain networks in response to placebo analgesia. For example, the activity of rostral ACC shows coupling with bilateral amygdalae and the PAG (Bingel et al., 2006; Eippert et al., 2009a), while a decrease in coupling was found between DLPFC and PAG (Sevel et al., 2015). Collectively, these findings suggest the involvement of the descending pain modulatory pathway, but the precise neural network and molecular mechanisms remain elusive. While the aforementioned studies used classical conditioning to induce placebo analgesia, recent research suggests that operant conditioning can also elicit the placebo effect (Janssens et al., 2019).

Several laboratories have attempted to generate an animal model for placebo analgesia, mainly using rodents via pharmacological conditioning, albeit with mixed results. Using morphine and other opioid agonists as unconditioned stimululi, both mice and rats have been shown to display responses resembling placebo analgesia (Miller et al., 1990; Valone et al., 1998; Bryant et al., 2009; Guo et al., 2010; Guo et al., 2011), while other studies have not (Nolan et al., 2012; McNabb et al., 2014; Akintola et al., 2019). In line with findings in humans, placebo analgesia in animals conditioned with opioids was reversed by opioid antagonists (Guo et al., 2010; Zhang et al., 2013). Further, in rats, placebo-like analgesia was inhibited by an antagonist specific to the µ-opioid receptor subtype in the rostral anterior cingulate cortex–a region implicated in human placebo responding (Petrovic et al., 2002; Wager et al., 2004; Zhang et al., 2013). Guo et al. (2010) also induced placebo-like responses in mice using a non-opioid NSAID, which was not blocked by naloxone. Overall, these animal studies demonstrate that certain aspects of placebo analgesia can be modeled in laboratory animals using classical conditioning and offer potential avenues to decipher pain memory mechanisms and the endogenous modulation of pain.

## Social Considerations

Sigmund Freud first observed that patients with pain problems tended to have family members with pain problems (Breuer and Freud, 1893). While this observation could be attributed to genetic relatedness, similar observations have been confirmed in genetically unrelated individuals living in the same household (Merskey and Spear, 1967; Turk et al., 1987). For instance, spouses of chronic pain patients suffer pain-related symptoms at a higher percentage than the spouses of people with diabetes (Flor et al., 1987b). Indeed, observer reinforcement of pain behavior has been assumed to play a significant role in shaping the severity and duration of chronic pain in the pain patient (Flor et al., 1987a). With this in mind, it is essential to recognize that pain occurs in a social sphere and is commonly communicated to and observed by others. This may start at a young age through children observing parents and other significant persons who teach them different attitudes about pain perception and responding to physical ailments (Baranowski and Nader, 1985). Viewed in this light, immediate family members with chronic pain may act as pain models to shape a child’s future pain behavior and experience.

One’s pain may be considered a personal experience, but it is rarely private and behavioral responses to pain function to communicate information to others within our social environment. The outward expression of pain universally indicates distress, which may elicit emotional reactions and caregiving actions from those around us. Humans communicate their pain experience through several different behaviors, including touching an injured body part, expressing discomfort through facial grimaces, and using vocal interjections such as “ouch.” Laboratory studies largely pioneered by Ken Craig and his colleagues have comprehensively documented the effect of social modeling and observation on psychophysical and psychophysiological responses to pain, finding that exposure of subjects to models exhibiting “tolerance” or “intolerance” to pain dramatically match the perceived level of tolerance (Craig and Weiss, 1971; Craig et al., 1975; Craig and Prkachin, 1978; Craig and Patrick, 1985; Goodman and McGrath, 2003). This is similar to results observed in rodents, where subjects match pain behavior and emotions when tested within a social environment.

**FIGURE 2:**
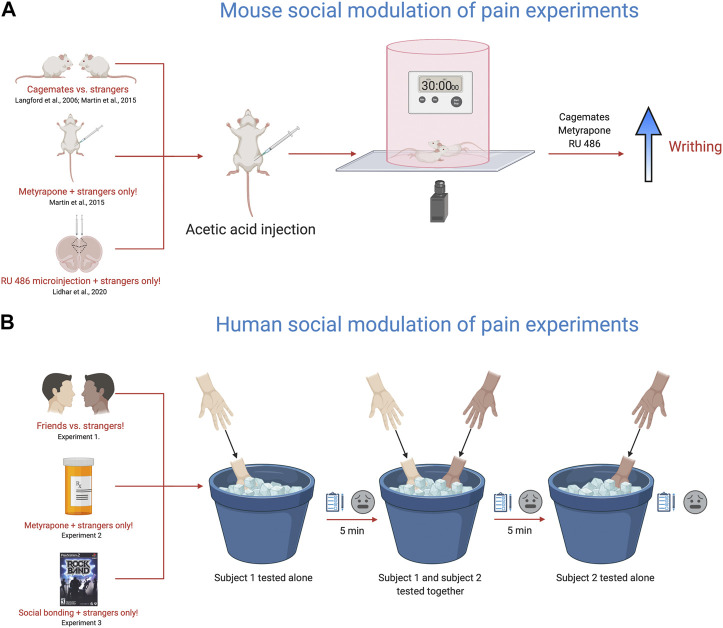
Translational behavioral models to test the influence of social context on pain responses. **(A)**. Using the acetic acid (0.9%) writhing test and manipulating social partner, drug pre-treatment or targeting specific brain areas, the nociceptive responses of mice are altered within the social environment. Placing two mice in a cylinder and injecting both mice with acetic acid (0.9%) enhances the nociceptive response of cagemates, but not strangers as originally reported in Langford et al., 2006. Further work with this model, showed that metyrapone, a glucocorticoid synthesis inhibitor recapitulated the cagemate effect in strangers, with metyrapone-injected stranger mice showing increased nociception (Martin et al., 2015). In addition, targeted injections of RU-486, a glucocorticoid receptor blocker also facilitated the nociceptive response of stranger mice (Lidhar et al., 2020). **(B)**. The cold pressor task was used to measure pain ratings in friends vs. strangers, strangers pre-treated with metyrapone and in strangers after engaging in a shared social experience (Martin et al., 2015). The experiment consists of three testing trials using the cold-pressor task. In the first test trial, subject 1 is tested alone by placing their non-dominant hand in the cold pressor for 30 s and then rating the pain intensity and unpleasantness using a visual analog scale. In the second trial, the second subject is brought into the room and both subjects are tested together by placing their non-dominant hand in the cold pressor for 30 s and then rating the pain intensity and unpleasantness using a visual analog scale. For the third trial, the second subject is tested alone by placing their non-dominant hand in the cold pressor for 30 s and then rating the pain intensity and unpleasantness using a visual analog scale. During trials where the participant is tested alone, the other subject is not present in the room. In calculating the overall pain ratings, the mean difference between a subject’s trial when tested alone was subtracted from their trial when tested with another participant. Friends tested together rated their pain higher than strangers, while metyrapone pre-treatment and playing the video game Rock Band (shared social experienced) enhanced the pain ratings in strangers. Overall, these models provide a new framework for studying the influence of social context on pain and offer insight into the fundamental mechanisms that engage the neural circuits responsible for pain modulation via social context. Given the complex nature of social context and social interactions on pain sensitivity in animals and people, dissecting their integral role in mediating pain outcomes is of critical importance.

The social modeling of laboratory pain in humans is strikingly similar to experiments performed in rodents, particularly mice (Figure 2A). When mice are tested in the presence of a familiar mouse also given a nociceptive stimulus, the nociceptive behavior of both mice is increased. This was first reported in 2006 and provided extensive evidence that nociceptive behaviors in mice are changed through social interactions and observation (Langford et al., 2006). Mice given a weak acetic acid injection into the stomach display twisting of the abdomen or writhing behaviors. Placing mice in an arena in dyadic pairings of cagemates enhanced the number of writhing episodes evoked by the acetic acid when compared with stranger dyads or mice tested alone. However, this enhancement was blocked by placing an opaque barrier between the cagemate pair of mice, suggesting that social observation was necessary for increased pain responses in cagemates (Langford et al., 2006). In the same study, when the target mouse was placed together with a mouse given a higher concentration of the inflammatory agent formalin, the target mouse licked the paw more compared to when paired with a mouse given the same concentration. In the reverse direction, when a target mouse was placed with a mouse given a weaker formalin concentration, the target mouse licked the paw less. This study showed a bidirectional modulation of nociceptive behavior, reminiscent of “tolerance matching” in the human social modeling experiments (Craig and Prkachin, 1978). While these findings may be explained by vicarious arousal or social modeling, they also may subserve processes related to empathy, as we’ve previously suggested (Martin et al., 2014; Sivaselvachandran et al., 2018a; Sivaselvachandran et al., 2018b).

It is conceivable that “pain communication” and “empathy” are synonymous concepts. Accordingly, our laboratory has been studying pain empathy in mice as a means to understand social-communicative pain behaviors (Martin et al., 2014; Sivaselvachandran et al., 2018a). The evidence for empathy in rodents shows that mice and rats consistently imitate arousal states and behaviors of one another; they will even sacrifice personal gain to relieve the distress of a fellow rodent (Langford et al., 2006, Jeon et al., 2010, Ben-Ami Bartal et al., 2011). In one line of research, we have been examining why unfamiliar (or stranger) mice do not show enhanced nociceptive responses when tested in each other’s presence. We came up with a relatively simple hypothesis–the social interaction of strangers is stressful, and this blocks the social facilitation of nociceptive responses, as observed in cagemates. To interrogate this hypothesis, we used a drug called metyrapone, which blocks the synthesis of cortisol and is a standard treatment for Cushing’s syndrome. To start, we pretreated all mice with metyrapone and tested mice in various social contexts (alone, cagemates, strangers) and dyadic pain status (i.e., only one mouse in pain or both mice in pain). In line with our hypothesis, stranger mice pretreated with metyrapone showed pain facilitation *on par* with cagemates (Figure 2A). Therefore, preventing glucocorticoid synthesis seemed to suppress the stress response induced by a stranger’s presence and ultimately promoted the display of empathic behavior in mice (Martin et al., 2015). Further, we have recently identified the prelimbic subdivision of the medial prefrontal cortex as an important node in controlling this behavior. Targeted microinjections of RU 486, a glucocorticoid receptor blocker into the prelimbic cortex enhanced nociceptive behavior in stranger dyads, but did not alter nociceptive responses of mice tested alone (Lidhar et al., 2020).

In support of our translational efforts, we designed a human experiment that paralleled our mouse experiments (Figure 2B). We recruited healthy participants to determine whether pain ratings were altered when subjects were tested in the presence of a friend or a stranger, similar to our mouse cagemate/stranger experiments. In line with our mouse findings, friends tested together–on the cold pressor task–reported greater intensity and unpleasantness than strangers (Martin et al., 2015). We then randomly divided a separate group of strangers into two groups and administered either 750 mg oral metyrapone or placebo, 60 min before cold pressor testing. Participants receiving metyrapone reported significantly increased pain intensity compared to those pretreated with placebo. These findings were congruent with our mouse experiments suggesting that blocking glucocorticoid synthesis increased pain sensitivity in strangers. Metyrapone also increased other non-verbal pain behaviors such as facial grimacing, handholding and rubbing when strangers were tested together. Finally, we conducted a third study and allowed participants–all strangers–to engage in a brief social gaming experience, where they bonded over the videogame RockBand. We found that stranger dyads who played RockBand together demonstrated increased stimulus intensity ratings, naturally alleviating the social stress induced by the mere presence of a stranger.

In Martin et al. (2015), we attempted an experiment where we administered intranasal oxytocin to participants, but pain responses were not enhanced when tested with a stranger. With regard to empathy and social communication, the neuropeptide oxytocin has received the most attention. It has been widely referred to as the *“love hormone”* because it modulates feelings of social attachment, trust, intimacy and empathy (Bartz et al., 2010; Hurlemann et al., 2010). Ultimately, our oxytocin experiment in humans did not work. We attempted similar–*unpublished*–experiments in mice using oxytocin antagonists to block the social facilitation of pain in cagemates, which also did not work. However, the pain modality and testing context may significantly influence oxytocin pain experiments and ratings. For instance, intranasal oxytocin increased the perceived pain intensity when subjects were asked to imagine that photographs of hands and feet in painful or nonpainful scenarios belonged to someone else (Abu-Akel et al., 2015). Interestingly, this same study showed that when participants were asked to imagine that the appendages were their own, oxytocin did not influence perceived pain intensity. There are also similar rodent experiments where oxytocin administration has been shown to enhance observational learning when a pain stimulus is delivered to another mouse (Pisansky et al., 2017). The observer mouse was administered intranasal oxytocin 30 min before watching another mouse receive a 0.8 mA of electric shock; this caused the observer mouse to exhibit profound freezing behavior, a common sign of fear in rodents. The effects of neuropeptides such as oxytocin are highly complex and as such we have not gone into great detail in this section.

We have previously reviewed the translational aspects of empathy in humans and mice including oxytocin (Sivaselvachandran et al., 2018b) and the studies mentioned here provide evidence for similarities between mouse and human subjects.

## Conclusion

The primary objective of the current review was to provide an overview of the translational approaches used in pain research, not necessarily clinical (pathological) pain. Of course, clinical pain is the problem, but understanding pain modulation and the relevant models of pain processing in healthy individuals is equally as important. For instance, one of the greatest risk factors for the development of chronic pain may be concurrent or past pain (Puntillo et al., 2001; Katz and Seltzer 2009), with the anxiety of impending pain crucial in determining whether pain sensitivity is enhanced (Ploghaus et al., 2001). Thus, studying pain-related behavior in healthy individuals in response to prior pain, trauma or social reinforcers are of physiological, and potential therapeutic value. Creatively developing animal models or testing similar phenomena in humans may also increase the validity of animal studies. These types of translational studies will enhance our understanding of pain perception and ultimately lead to improved treatment methods.

Rarely have behavioral pain experiments compared pain modulation using animals and humans in a single paper. In the translational studies that we have conducted, all experiments were performed in-house or in close collaboration with other labs, where the primary author designed and directed all research. Collaboration was especially important because there are considerations for human research that must be taken into account that are not so obvious for rodent researchers and vice versa. While we recognize that it may not always be possible to directly compare multiple species for every paper, a more concerted effort should be made within this domain. Conducting behavioral experiments within a single lab (or with the same personnel) allows for the most optimal experiments to be designed and allows for efficient troubleshooting of procedural problems so that researchers can assess whether translation does or does not exist, or a methodological problem persists. Once translation is suspected to be possible (or not), initial findings can then be replicated and extended by other labs. It is prudent to point out that many investigators may refrain from implementing such an approach in their laboratories because these experiments are laborious, high focus, and require significant numbers of staff to perform. However, where possible we believe that the development of novel translational models to assess pain behavior should be prioritized as they are not technically challenging and may be performed by adequately trained undergraduate researchers. From a behavioral perspective, having mouse and human experiments conducted in parallel is an exciting approach for the initial assessment of model relevancy and species translation.

There exists a diversity of available animal models of human chronic pain but understanding what animal models are relevant to human pain modulation is essential to provide more information about the development of chronic pain. To fully understand pain phenotypes and modulation, a full battery of assays, including multiple modalities and injury types, should be considered (Mogil et al., 2006), and because various artifacts can confound some but not other assays (Callahan et al., 2008). Newer approaches, such as operant techniques (Murphy et al., 2014) or measuring facial expressions of pain (Langford et al., 2010), might be beneficial adjuncts to the standard current batteries. However, most of the papers that use these assays do not conduct conceptually similar experiments in humans. Assessment of facial pain expressions may be a highly valuable translational tool, as pain faces are evolutionarily conserved and would allow for similar modalities of assessment using analogous metrics in animals and humans (Chambers and Mogil 2015).

The reliance on current mouse models, especially inbred mice may not be appropriate for modeling complex human conditions with the explicit purpose of clinical translation. The individual differences commonly observed with human pain conditions are difficult to reproduce with the inbred homogeneous animal populations, particularly genetic and environmental variability (and their interaction). While inbred mice have been preferentially selected (and even necessary) for molecular genetic studies, the strong preference for inbred subjects derives from inertia and the assumption that outbred stocks would amount to increased phenotypic variation and necessitate the use of higher sample sizes. This is certainly a reasonable assumption; however, it is not supported by the current evidence since the coefficient of variability comparing phenotypic outcomes between inbred and outbred populations are similar (Jensen et al., 2016; Tuttle et al., 2018). While mechanistic studies are hugely important for the development of novel therapies and offer a complete understanding for the basis of disease, they may be of little use if those therapies or mechanisms do not translate across species. In this regard, variability in laboratory (i.e., environmental) conditions may improve phenotypic heterogeneity to better capture the variability observed by human disease populations. These include differences in food type, bedding material, cage size, humidity, temperature, light cycles and identification method (Richter et al., 2011). Thus, we are suggesting that the use of heterogeneous animal—especially mouse—populations may provide a better way forward in assessing the generalizability of results across experimental conditions where treatments—especially pharmacological—are the goal.

Aside from rodent research, using populations of domestic animals that display naturally occurring diseases may also be a potential future direction of pain studies. Many domesticated animals display pathophysiological similarities to human pain conditions including bone cancer-related pain and degenerative joint disease (Kol et al., 2015; Klinck et al., 2017). Examining these naturally occurring conditions in various animal species may be part of the solution for conducting effective translational research. Domesticated animals are genetically diverse and are subject to varying environmental conditions. Many domesticated animals are treated like family and are exposed to similar environmental conditions as humans. Watkins et al., 2020 recently showed that a novel DNA-based therapy was well-tolerated, safe and effective for the treatment of advanced osteoarthritis in companion dogs. Limiting the use of companion animals to the sphere of randomized control trials would offer a distinct advantage for the discovery and validation of novel therapies for the treatment of naturally occurring pain conditions such as arthritis and cancer-bone pain (Kol et al., 2015); this would offer a giant step forward. Of course, the widespread use of companion animals would be met with heightened sensitivity and ethical issues, but their inclusion has the added benefit of potentially finding therapies for our most beloved pets as well as chronic pain patients. The inclusion of domesticated animals may increase the validity of findings to offer a higher degree of translational success.

In chronic pain management, our goal is to treat the individual as best we can; however, factors such as experience with pain, psychological profile, and the social environment cannot be accounted for in the same manner as biological targets. These factors drastically alter pain responses and the way we treat individual pain patients, especially when they override or interact with drug treatments. Conclusions drawn from various translational studies have successfully shown the influences of empathy, memory, and individual differences on subjective pain experiences. Animal studies are often criticized for the lack of biological validity, but we have had the most success in developing cognitive and social modulation of pain experiments that translate between species. By focusing a portion of future pain research into developing highly translational pain models, we may drive the enhancement of pain therapeutics and conclusions drawn from animals to humans.
